# Pulsed UV laser light on *Escherichia coli* and *Saccharomyces cerevisiae* suspended in non-alcoholic beer

**Published:** 2011-03

**Authors:** SM Hosseini, MK Azar-Daryany, R Massudi, A Elikaei

**Affiliations:** 1 Assistant Professor, Department of Microbiology, Faculty of Biological Sciences, Shahid-Beheshti University, Evin, 19839, Tehran, Iran; 2 Department of Medicine, The University of Sydney, NSW, Australia; 3 Laser Research Institute, Shahid Beheshti University, Evin 19839,Tehran, Iran

**Keywords:** Pulsed UV laser light, Inactivation, *E. coli*, *S. cerevisiae*, non-alcoholic Beer

## Abstract

**Background:**

The aim of this study was to investigate the effect of pulsed ultra-violet (UV) irradiation on inactivation of beer spoilage microorganisms. UV irradiation is nowadays cost effective enough to compete with traditional biological, physical, and chemical treatment technologies and has become an alternative to such methods.

**Material and Methods:**

Photoinactivation effects of pulsed UV laser with the wavelengths of 355 and 266 nm, which inactivate typical prokaryotic (*Escherichia coli*) and eukaryotic (*Saccharomyces cerevisiae*) microorganisms, were examined with different doses and exposure times.

**Results:**

A dose of 100 J/cm^2^ of the 355 nm pulsed UV laser was able to reduce about 1 to 2 log (88.75%) of *E.coli* with the population of 1.6×10^8^ colony-forming units (CFU/ml), and 97% of 3.2×10^7^, 3×10^6^, 5.5×10^5^, and 9×10^4^ CFU/ml. In the case of 266 nm, more than 99% reduction in *E. coli* serial dilutions was inactivated, using 10 J/cm^2^ with exception of 7×10^4^ CFU/ml which was not detected any bacterial growth using 5 J/cm2. In addition, 50, 40, and 20 J/cm^2^ energy were used successfully to inactivate *S. cerevisiae* at the populations of 5.4×10^6^, 7×10^5^, 5×10^4^ and 4×10^3^ CFU/ml, respectively. As a result, pulsed UV Laser with 266 nm was strong enough to inactivate a high titer of bacterial and yeast indicator standards suspended in non-alcoholic beer in comparison with 355nm doses.

**Conclusion:**

Results indicate that pulsed UV technology, in principle, is an attractive alternative to conventional methods for the inactivation of indicator microorganisms and has potential in irradiation of unpasteurized beer.

## INTRODUCTION

The effects of contamination range from comparatively minor changes in beer flavor and fermentation performance to gross flavor defects and superattenuation of worts (1). Beer is a relatively hostile environment to many microorganisms. The antiseptic properties of hop compounds are increased by ethanol. Low redox and acid pH provide additional protection against many potential spoilage microorganisms. Ethanol is a powerful inhibitor of microbial growth, but low and zero alcohol beers have a much increased susceptibility to spoilage compared to their alcoholic counterparts. Several bacterial and some yeast species are capable of growth in beer. This can cause the formation of hazes, surface pellicles and many undesirable changes in beer flavor and aroma (1).

Sterility is traditionally achieved by pasteurizing the beer in the bottle after filling, (tunnel pasteurization), or before filling (flash pasteurization or sterile filtration) (2). A new sterilization technique based on the use of pulsed UV irradiation is suggested to have great potential in the development of a new method of sterilization (3). In addition, FDA has given premarket approval to use UV radiation for the treatment of water and food including juices, and to achieve a 5-log reduction in numbers of the most resistant microorganisms, under specific conditions of use (4–6).

In this study, the photoinactivation property of pulsed UV laser radiation at wavelengths of 355 and 266 nm, used as a physical means to inactivate two typical microorganisms, prokaryotic (*Escherichia coli*) and eukaryotic (*Saccharomyces cerevisiae*), with respect to dose and exposure times, was examined. Furthermore, the effectiveness of UV laser treatments with respect to their doses and exposure times was determined.

## MATERIAL AND METHODS

**Laser operation**. A Q-switched Nd:YAG laser (NL 301G, EKSPLA) was used for experimental realization. The diameter of the beam was 6 mm, and pulse duration was 5 ns, with a repetition rate of 10 Hz, which comes with third and fourth harmonics; 355 nm, 266 nm. The output energy of the laser at the third and fourth harmonics, adjusted to 80% of its maximum energy, were 60 & 10 mJ/pulse, respectively.

**Preparation of Test Microorganisms Suspension**. *Escherichia coli* (K12) was grown under optimal conditions in Nutrient broth (Merck), at 37°C, in a shaker to ensure sufficient cell density. An 18 hour culture was used for experimental purposes to mimic environmental conditions. The cell suspension was centrifuged and the supernatant removed. The pellet was resuspended in non-alcoholic beer (Behnoosh Inc. Iran) to obtain an *E. coli* concentration of approximately 1.5×108 Colony- Forming Unit (CFU/ ml) as determined by 0.5 McFarland standard and spectrophotometric assays. Serial dilutions of 108, 107, 106, 105, and 104 CFU/ml of tested microorganisms were prepared. The samples were mixed thoroughly, and 150 µl of each dilution was poured into sterilized quartz tubes for radiation experiments. After exposure, 100 µl of irradiated and nonirradiated (control) cells were cultured on MacConkey Agar (Merck). CFU number was determined by pour plate method after incubation at 37°C for 72 hours.

*Saccharomyces cerevisiae*, wild-type (commercial bakery yeast) strain, was cultivated in yeast extractpeptone-dextrose broth at 37°C for 14 to 18 hours. The cell suspension was centrifuged and pellet resuspended in non-alcoholic beer to obtain a concentration of approximately 1.5×106 CFU/ml as determined by 0.5 McFarland standard and spectrophotometric assays. Serial dilutions of 106, 105, 104, and 103 CFU/ ml were used for the irradiation experiment. Treated and untreated samples were then plated on peptone dextrose agar (Merck). CFU number was determined by pour plate method after incubation for 72 hours at 37°C.

**Fig. 1:**
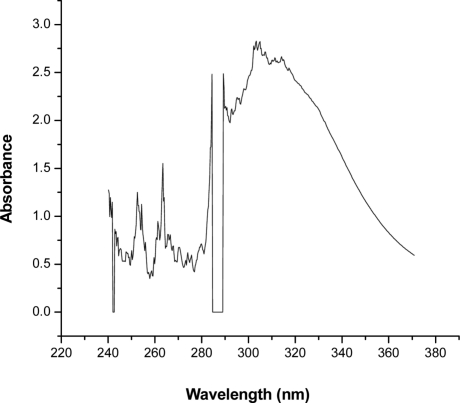
Non-alcoholic beer absorption in the UV region, Distilled water was adjusted as a blank sample in order to obtain beer absorption.

**Sample irradiation**. Quartz tubes with an inner diameter of 5 mm were used for irradiation of microorganisms. Absorption of non-alcoholic beer was obtained by spectrophotometer (Ocean Optics HR4000, Florida, USA) in the UV region, which was 0.753 at the 266 nm and 0.962 at the 355 nm wavelength (Fig. 1). Continuous stirring kept the cells suspended and obtained good homogeneity, and the speed of the magnet was well adjusted to avoid turbidity.

A laser beam, 6-mm diameter, was adjusted to equal the height of the cell suspension in the quartz tubes to cover the entire solution. Samples were exposed UV radiation, 0 to 100 J/cm2, at room temperature (20–25°C), and the repetition rate was 10 Hz.

The dose for each microorganism was determined by maintaining exposure intensity while varying exposure time. For positive controls, identical samples were incubated for the duration of the exposure time at room temperature. Concentration of test microorganisms were expressed as CFU/ml in log-10 manner and viable organisms were counted before and after irradiation. Exposed and non-exposed samples were maintained at 4°C until enumeration. The time intervals between completion of irradiation and incubation of microorganisms were<20 minutes. Each experiment was repeated three times, and the average values are presented in the results section.

**Statistical Analysis**. Each experiment was performed three times, and the average and SD of the mean are presented for each sample. Statistical analysis (general linear model analysis of variance [ANOVA]) was performed using SPSS software (standard version 9.0; SPSS, Chicago, IL) for each experimental condition.

## RESULTS

**Fig. 2:**
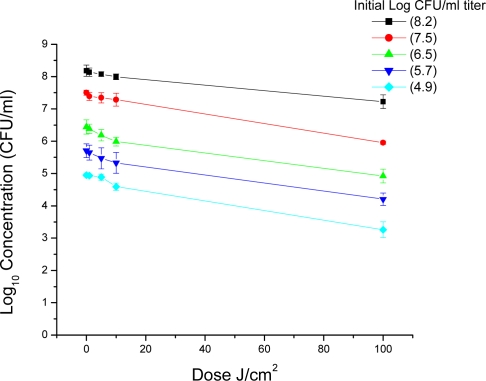
Log_10_ CFU/ml survival curves of *E. coli* at 355-nm wavelength of pulsed UV laser.

**Fig. 3:**
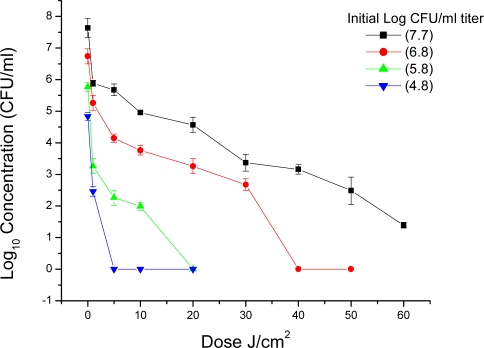
Log_10_ CFU/ml survival curves of *E. coli* at 266-nm

**Fig. 4:**
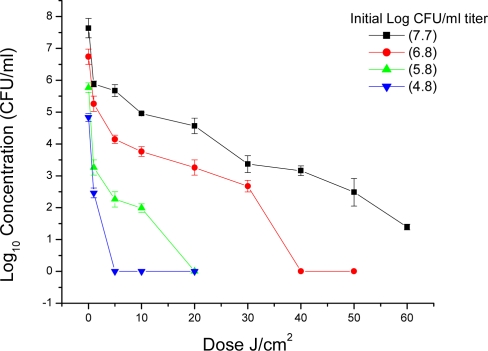
Log_10_ CFU/ml survival curves of *S. cerevisiae* at wavelength of pulsed UV laser. 266-nm wavelength of pulsed UV laser.

The log reductions (survival curve) for each microorganism were plotted as a function of pulsed UV dose for each wavelength and are presented in Figs. (2–4).

***E. coli*** **Inactivation Curves**. As shown in Fig. 2, a dose of 100 J/cm2 of the 355 nm pulsed UV laser was able to reduce an *E. coli* population of 1.6×108 CFU/ml by about 1 log (88.75%), and this decrease was increased to about 2 log (97%) for populations of 3.2×107, 3×106, 5.5×105, and 9×104 CFU/ml. However, using similar doses, higher inactivation rates in different populations could not be achieved. Independent of cell density and when using 10 J/cm2 energy, more than 50% reduction in cell numbers was observed in bacterial populations of 3×106, 5.5×105, and 9×104 CFU/ml. Nonetheless, the log reductions at higher cell densities were not the same as above results and remained at 37.5%.

As shown in Fig. 3, when using 266-nm wave- length, and a dose of 10 J/cm2, more than 3 log inactivation was detected in *E. coli* 5×107, 6×106, and 6×105 cell numbers, however, in cell density of 7×104 CFU/ml no bacterial growth was observed using 5 J/ cm2 energy dose. A 5 log reduction (99.999%) of *E. coli* with population of 5×107 was achieved with the dose of 50 J/cm2. Other *E. coli* populations, 6×106 and 6×105 CFU/ml were completely inactivated using 40 and 20 J/cm2, as no bacterial growth was observed in pour plate method after 72 hours.

***S. cerevisiae*** **Inactivation Curves**. Due to the fact that irradiation of *E. coli* at 355 nm was not able to inactivate the 5-log of bacterial population, in reference to new rules which are needed (6–8), irradiation of *S. cerevisiae* at 355 nm wavelength was not performed further.

As shown in Fig. 4, a 30 J/cm2 energy dose at 266 nm resulted in 4 log of inactivation of *S.cerevisiae* at cell densities of 5.4×106 (99.999%), 7×105 (99.99%), and 5×104 (99.97%) CFU/ml. Increasing the energy dose to 20, 40, and 50 J/cm2 resulted in full inactivation of *S. cerevisiae* population at cell density of 5.4×106, 7×105, and 5×104 CFU/ml yeast, respectively.

However, a 20 J/cm2 energy dose of pulsed UV killed all cells at a density of 4×103 CFU/ml. General linear model ANOVA was employed to statistically analyze the results, which indicated that the tested microorganisms showed significant effects for different dose levels and within different populations; however, when microorganisms exposed to low-dose levels, i.e., 355 nm (≤ 10 J/cm2), the inactivation rates were not significant. The inactivation rates at 266-nm wavelength were statistically significant both within and between different doses and microorganism populations (P ≤ 0.05).

## DISCUSSION

UV processing involves the treatment of foods with radiation from the UV region of the electromagnetic spectrum to inactivate microorganisms. UV treatments have been applied effectively to water supplies and food contact surfaces (9), and to apple juice (10–12). Pulsed UV has a higher penetration depth and may be more effective than continuous UV light (13).

Monochromatic pulsed UV has been shown to inactivate bacteria in milk (14, 15), however, its effectiveness against the target microorganism for traditional pasteurization methods has not been evaluated in such fluids e.g., milk, beer, and fruit juices, so, equivalence to thermal pasteurization has not been known properly (6).

In this study, the pulsed UV irradiation effects on *E. coli* and *S. cerevisiae* were examined. A 355 nm laser dose of 100 J/cm2 was sufficient to inactivate *E. coli* population of 3.2 ×107 CFU/ml by more than 1 log. Moreover, in parallel experiments, a lower-energy dose (60J/cm2) at 266 nm wavelength significantly inactivated a larger population of *E. coli* (5×107CFU/ ml) by more than 6- log. In addition, a lower population of *E. coli* was completely inactivated when an even lower energy dose (40 J/cm2) was employed at 266 nm. Also, other *E. coli* populations (6×105 and 7×104 CFU/ml) were inactivated completely by 10 and 5 J/ cm2, respectively. These results are in agreement with previous studies (3, 16–18) using pulsed light. In this case, inactivation of *E. coli* was achieved at around 270 nm, whereas wavelengths above 300 nm were inefficient for inactivating *E. coli*. Also, the most significant and effective inactivation of *S. cerevisiae* at 5.4×106 CFU/ml was observed at 266 nm and 50 J/ cm2 energy dose. Other populations of *S. cerevisiae* (7×105, 5 ×104, and 4×103 CFU/ml), were completely inactivated with energy doses of 40, 40, and 20 J/cm2, respectively. In these studies, a 7-log concentration of *E. coli* is almost similar to a 5- log concentration of *S. cerevisiae*. The difference in population between these two microorganisms might have been caused by their sizes because *S. cerevisiae* is 100 times larger than *E. coli*. The maximum population of the test microorganisms in this study was designed to be greater than that of untreated food, beverages, and running water. Therefore, the irradiation protocols outlined in this study were designed for the worst case.

The laser employed in our experiment was operated in pulse mode. It has been shown that high- peak pulse power has irreversible effects on DNA and produces active species, which can damage the genome and other intracellular components, causing lethal and sublethal effects (3, 14, and 15, (19, 21, 22). In contrast, Ougoma et al. (19) reported that the time required for photoreactivation is 1 to 3 hours. However, in our study, irradiation time was less than 10 minutes. Therefore, it could be concluded that the photoinactivation process in our study could not be revoked.

The data which is obtained from irradiation of *E. coli* and yeast indicate that, irradiation effect on yeast was not as dramatic as the effect on *E. coli*. As mentioned by Vasilenko 2001 (23), this is good for the commercial application on the laser treatment. The laser treatment can be used on all kind of beers that are not normally pasteurized or sterilized because the laser irradiation will not greatly affect yeast viability but will kill bacteria. So, it is important to have some living yeast in bottle conditioned beer to continue natural fermentation (23).

In summary, pulsed UV disinfection has many advantages over alternative methods. Unlike chemical biocides, UV does not introduce toxins or residues and does not alter the chemical composition, taste, odor or pH of the fluid being disinfected. This feature is especially important in the brewing and beverage industries where the beer needs to be kept free from contamination by gram negative bacteria, which can cause off-flavors and acidity and alter the chemical properties of the product or, in the case of brewing specifically, affect the fermentation process.
